# Abnormal EEG Complexity and Functional Connectivity of Brain in Patients with Acute Thalamic Ischemic Stroke

**DOI:** 10.1155/2016/2582478

**Published:** 2016-06-14

**Authors:** Shuang Liu, Jie Guo, Jiayuan Meng, Zhijun Wang, Yang Yao, Jiajia Yang, Hongzhi Qi, Dong Ming

**Affiliations:** ^1^Neural Engineering & Rehabilitation Lab, Department of Biomedical Engineering, College of Precision Instruments and Optoelectronics Engineering, Tianjin University, Tianjin 300072, China; ^2^Department of Neurology, Tianjin First Center Hospital, Tianjin 300072, China

## Abstract

Ischemic thalamus stroke has become a serious cardiovascular and cerebral disease in recent years. To date the existing researches mostly concentrated on the power spectral density (PSD) in several frequency bands. In this paper, we investigated the nonlinear features of EEG and brain functional connectivity in patients with acute thalamic ischemic stroke and healthy subjects. Electroencephalography (EEG) in resting condition with eyes closed was recorded for 12 stroke patients and 11 healthy subjects as control group. Lempel-Ziv complexity (LZC), Sample Entropy (SampEn), and brain network using partial directed coherence (PDC) were calculated for feature extraction. Results showed that patients had increased mean LZC and SampEn than the controls, which implied the stroke group has higher EEG complexity. For the brain network, the stroke group displayed a trend of weaker cortical connectivity, which suggests a functional impairment of information transmission in cortical connections in stroke patients. These findings suggest that nonlinear analysis and brain network could provide essential information for better understanding the brain dysfunction in the stroke and assisting monitoring or prognostication of stroke evolution.

## 1. Introduction

Stroke is a leading cause of disability with a relatively high death ratio around the world, and up to 70% of stroke patients are left with moderate or severe functional impairment [1], placing a heavy physical and mental burden on both the patients and their families. It has been reported that early rehabilitation after stroke can enhance recovery, minimize functional disability, and reduce potential costly long-term care expenditures [1]. Patients with thalamic stroke only show minor changes on physical examination; thus valid and objective early diagnosis becomes extremely necessary.

The rapid development of imaging technology, such as computed tomography (CT), positron emission tomography (PET), magnetic resonance imaging (MRI), and electroencephalography (EEG), gives researchers an access to an embedded knowledge of the brain changes during stroke onset and recovery process [2]. Among these, EEG offers a continuous, real-time, and noninvasive measure of brain function, providing new insights into poststroke cerebral pathophysiology [3–6]. Because of its sensitivity to metabolic and ionic disturbances related to ischemia, it can be a potentially useful tool for acute stroke detection and for monitoring affected tissue [6]. In acute ischemic stroke, the primary injury has typically occurred prior to presentation, but EEG may be able to detect patterns to suggest severity, prognosis, and secondary injury (e.g., reocclusion, edema, or hemorrhagic transformation) [7]. Several parameters have been found correlated with initial stroke severity as measured by the National Institutes of Health Stroke Scale (NIHSS) in both the acute (brain symmetry index) [8, 9] and subacute periods (relative alpha percentage, relative alpha-beta percentage, relative delta-theta percentage, delta/alpha ratio, delta-theta/alpha-beta ratio, and global pairwise derived BSI) [10–12]. In summary, the existing researches mostly concentrated on power spectral density (PSD), and the PSD in various frequency bands has revealed to be a distinguishable indicator [13].

It is well accepted that brain is truly a complex system; therefore nonlinearities and nonlinear measures must be taken into account in its modeling and analysis [14, 15]. It has been reported that nonlinear parameters were more sensitive to both the power spectrum and the temporal amplitude distribution comparing with conventional spectral analysis in many other diseases [16–19]. Consequently, nonlinear analysis of the EEG may shed additional light on analyzing cortical information processing deficits in acute thalamic ischemic stroke. The first nonlinear methods that were used to analyze EEG are the correlation dimension (D2) and the first Lyapunov exponents (L1). D2 was applied by Grassberger and Procaccia in 1983 to quantify the number of independent variables that are necessary to describe the dynamic system. It was used to provide the statistical characteristic of the system. By contrast, L1 was applied by Wolf in 1985 as a dynamic measure to gauge the flexibility of the system [20, 21]. Then several methods of complexity and entropy techniques have dealt with the complexity or irregularity in the ability of the system to create information and showed promising results in detecting EEG abnormalities [14]. On-line use of D2 and L1 in a clinical situation is still impractical, since reliable estimation of them requires a large quantity of data and a long calculation time. Fortunately, the Lempel-Ziv complexity (LZC) measure [22] can act as an alternative tool for EEG analysis, since it is well suited for characterizing the development of spatiotemporal activity patterns in high-dimensionality nonlinear systems, like brain and heart. Moreover, the concept of LZC is simpler to understand and its computation is easier to implement. Entropy is a concept addressing randomness and predictability, with greater entropy often associated with more randomness and less system order [23]. Among these, Sample Entropy (SampEn) [24] has stood out with excellent antinoise and anti-interference performance; moreover, it could predict stable values with shorter data. LZC and SampEn have shown encouraging results in differentiating patients and healthy controls in some mental diseases, such as Alzheimer's disease [25], Schizophrenia and depression [18], and global Hypoxic-Ischemic brain injury [26].

More recently, resting-state functional connectivity has a strong genetic component and shows characteristic changes in various psychiatric and neurological disorders [27]. Functional connectivity is assumed to reflect functional interactions between the underlying brain regions and has become very popular in the study of brain mechanisms underlying disturbed cognition diseases such as Alzheimer's disease and Parkinson's disease [28–30]. For the stroke, loss of neurons, as well as disturbed synaptic transmission, may lead to abnormal functional interactions between cortical regions.

In this study, we recorded the resting-state EEGs in patients with thalamic ischemic stroke from the acute stage and controls. Nonlinear analysis and functional connectivity were firstly employed to discriminate between the strokes and controls, providing a new insight into the brain changes in the stroke patients. LZC, SampEn, and partial directed coherence (PDC), which are important but always overlooked methods in stroke studies, were used to calculate the EEG features in order to seek and determine the proper and distinguishable way in the diagnosis of acute thalamic ischemic stroke disease.

The remainder of this paper is structured as follows.  Section 2 describes the experimental methodology and systematically details signal processing methods. Contrastive analysis on EEG features between strokes and controls is addressed in Section 3. At last, the discussion and conclusion are stated in Sections 4 and 5, respectively.

## 2. Materials and Methods

### 2.1. Subjects

From December 2012 to January 2015, 12 patients (7 females and 5 males; mean age of 65 years) with ischemic thalamic stroke took part in the study, with 11 healthy subjects (4 females and 7 males; mean age of 53 years) as controls. Independent-samples *t*-test were performed on ages between two groups, and there were no statistical differences (*t* = −1.749, df = 12.828, and *p* = 0.104). The clinical data were acquired in Tianjin First Central Hospital. Inclusion criteria of the patients consisted of a focal ischemic lesion of the thalamus and a symptom of hand numbness. Exclusion criteria were a history of substance abuse, additional neurological or psychiatric disorders, and current psychoactive drug treatment. All the patients had been examined from the acute stage (<7 days).

### 2.2. EEG Recording

EEG was recorded with the Netlink 40 developed by the Bio-logic company. The 10–20 system of electrode placement was used with electrodes placed at Fp1, Fp2, F3, F4, F7, F8, Fz, T3, T4, T5, T6, A1, A2, C3, C4, Cz, P3, P4, Pz, O1, O2, and Oz. The sampling rate was 256 Hz and impedance was kept below 10 k′Ω. The EEG was recorded in the resting condition with eyes closed. The raw EEG data were exported into MATLAB (The MathWorks, Natick, MA, USA) for further analysis.

### 2.3. Calculation of EEG Features

#### 2.3.1. Preprocessing

Prior to further analysis, a preprocessing stage of the EEG signals is required. Five-minute data without apparent artifacts (such as blinks, EMG, and visible drift) were selected manually from each patient's EEG recording. All channels were rereferenced to bilateral mastoid, band pass filtered at 1–45 Hz. EEG data were then split into five-second, nonoverlapping epochs in the following step of feature extraction. 60 epochs of artefact-free EEG data per participant was obtained finally.

#### 2.3.2. Lempel-Ziv Complexity (LZC)

An algorithmic complexity measurement introduced by Lempel and Ziv [22] is a measure reflecting the rate of new pattern generation along given sequence of symbols. In other words, it characterizes the structure or, as the name implicates, the complexity of the signal whether the signal is predictable (has simple structure) or nonpredictable (has complex, random structure) [31]. Applications of Lempel-Ziv complexity (LZC) to EEG signals include assessment of the depth of anesthesia [32] and sedation [31] and analysis of the background activity in Alzheimer's disease [33].

The calculation algorithm of LZC for the sequence of symbols *X*_1_^*N*^ = *x*_1_, *x*_2_, *x*_3_,…, *x*_*N*_ of length *N* is defined as follows. A block *B* of length *k* (1 ≤ *k* ≤ *N*) is a subsequence of *k* consecutive symbols, *B* = *x*_*i*+1_^*i*+*k*^ = *x*_*i*+1_, *x*_*i*+2_,…, *x*_*i*+*k*_ (0 ≤ *i* ≤ *N* − *k*). The first block, *B*_1_, is set equal to the first symbol of the sequence *x*_1_^*N*^; that is, *B*_1_ = *x*_1_^1^ = *x*_1_. Next

(1) $$\begin{array}{c} B_{d+1}=x_{n_{d}+1}^{n_{d+1}},\;\left(n_{d}+1\leq n_{d+1}\leq N\right) \end{array}$$

is defined to be the following consecutive block of minimal length such that it does not occur in the sequence *x*_1_^*n*_*d*+1_−1^. Therefore, by continuing this recursive procedure until the last symbol of *x*_1_^*N*^ is reached it is possible to obtain the decomposition of *x*_1_^*N*^ into minimal blocks

(2) $$\begin{array}{c} x_{1}^{N}=B_{1},B_{2},\ldots ,B_{n}. \end{array}$$

The complexity Ca of *x*_1_^*N*^ is defined as the number of blocks in the decomposition, *n*(10)

(3) $$\begin{array}{c} c_{\alpha }\equiv n=n\left(\;\alpha \;\right), \end{array}$$

where *α* is the number of possible different symbols in *x*_1_^*N*^. The normalized complexity, *C*_*α*_, is defined as

(4) $$\begin{array}{c} C_{\alpha }=\frac{c_{\alpha }\left(x_{1}^{N}\right)}{N/\log_{\alpha }N}=\frac{n\left(\alpha \right)}{N}\log_{\alpha }N. \end{array}$$

Prior to applying the above described algorithm the signal *s* has to be converted into a sequence of symbols, which can be done as follows. Depending on the number of different symbols *α*, *α* − 1 thresholds *T*_*i*_ have to be selected within the signal range, *s*_min_ < ⋯<*T*_*i*_ < ⋯<*s*_max_, where *s*_min_ and *s*_max_ are the minimum and maximum values of the signal *s*, respectively. For instance, if *α* = 2, that is, two symbols, “0” and “1” are used, there is only one threshold *T*_1_ and by comparing the samples of *s* with this threshold the signal is converted into the sequence of symbols: if *s*(*i*) < *T*_*i*_ then *x*_*i*_ = 0; otherwise *x*_*i*_ = 1. For larger *α*, the conversion procedure is analogous. Here, the median voltage was considered as the threshold.

LZC values were also calculated in different frequency bands, including delta (1–4 Hz), theta (4–8 Hz), alpha (8–13 Hz), and beta (13–30 Hz).

#### 2.3.3. Sample Entropy (SampEn)

The name of SampEn refers to the applicability to time series data sampled from a continuous process [24]. SampEn does not use a templatewise approach when estimating conditional probabilities. It only requires that one template find a match of length *m* + 1 and then computes the logarithm of a probability associated with the time series as a whole. It is well known that entropy is a measure of the rate of information generation; a larger SampEn presents lower self-resemblance and a higher rate of information generation of the signal. Mathematically, to compute SampEn we follow the steps explained as below.

Given a signal *X* = (*x*_1_, *x*_2_,…, *x*_*N*_), where *N* is the total number of data points, SampEn algorithm [34] can be summarized as follows.

(1) Form a set of vectors *X*_*m*_^1^,…, *X*_*m*_^*N*−*m*+1^ defined by

(5) $$\begin{array}{c} X_{m}^{i}=\left(\;x_{i},x_{i+1},\ldots ,x_{i+m-1}\;\right),\;i=1,\ldots ,N-m+1. \end{array}$$

(2) Define the distance between vectors *X*_*m*_^*i*^ and *X*_*m*_^*j*^ as the maximum absolute difference between their respective scalar components:

(6) $$\begin{array}{c} d\left[\;X_{m}^{i},X_{m}^{j}\;\right]=\underset{k=0,\ldots ,m-1}{\max}\left|\;x_{i+k}-x_{j+k}\;\right|. \end{array}$$

(3) For a given *X*_*m*_^*i*^, count the number of *j* (1 ≤ *j* ≤ *N* − *m*,  *j* ≠ *i*), denote as *B*_*i*_, such that *d*[*X*_*m*_^*i*^, *X*_*m*_^*j*^] ≤ *r*, that is, *B*_*i*_ is the number of *d*[*X*_*m*_^*i*^, *X*_*m*_^*j*^] ≤ *r*, *j* ≠ *i*. Then, for 1 ≤ *i* ≤ *N* − *m*,

(7) $$\begin{array}{c} B_{i}^{m}\left(\;r\;\right)=\frac{1}{N-m-1}\times B_{i}. \end{array}$$

(4) Define *B* ^*m*^(*r*) as

(8) $$\begin{array}{c} B^{m}\left(\;r\;\right)=\frac{1}{N-m-1}B_{i}. \end{array}$$

(5) Similarly, calculate *A*_*i*_^*m*^(*r*) as 1/(*N* − *m* + 1) times the number of *j* (1 ≤ *j* ≤ *N* − *m*,  *j* ≠ *i*), such that the distance between *X*_*m*+1_^*j*^ and *X*_*m*+1_^*i*^ is less than or equal to *r*:

(9) Aimr=1N−m−1×no.  of  dXm+1i,Xm+1j≤r,i≠j.

Set *A* ^*m*^(*r*) as

(10) $$\begin{array}{c} A^{m}\left(\;r\;\right)=\frac{1}{N-m}\overset{N-m}{\underset{i=1}{\sum }}A_{i}^{m}\left(\;r\;\right). \end{array}$$

Thus, *B* ^*m*^(*r*) is the probability that two sequences will match for *m* points, whereas *A* ^*m*^(*r*) is the probability that two sequences will match for *m* + 1 points.

(6) Finally, define

(11) $$\begin{array}{c} \text{SampEn}\left(\;m,r\;\right)=\underset{N\to \infty }{\lim}\left\{\;-\ln\left[\;\frac{A^{m}\left(r\right)}{B^{m}\left(r\right)}\;\right]\;\right\}. \end{array}$$

Which is estimated by the statistic

(12) $$\begin{array}{c} SampEn\left(\;m,r,N\;\right)=-\ln\left(\;\frac{A^{m}\left(r\right)}{B^{m}\left(r\right)}\;\right). \end{array}$$

As can be seen in the equations described above, *m* and *r* are the two parameters that must be specified for the calculation of SampEn. Pincus and Goldberger [35] have suggested that *r* be set between 0.1 and 0.25 times standard deviation of the signal and *m* be set equal to 1 or 2. In this work, we set *m* = 2, *r* = 0.2. SampEn values were also calculated in different frequency bands, including delta (1–4 Hz), theta (4–8 Hz), alpha (8–13 Hz), and beta (13–30 Hz).

#### 2.3.4. Partial Directed Coherence

The functional connectivity was estimated with partial directed coherence (PDC) [36]. PDC is based on the concept of partial coherence, a technique that quantifies the directed interdependence of Granger causality between any two signals in a multivariate set. A detailed description of PDC along with an illustrative example is given by Baccalá and Sameshima [37]. Hence, the mean values within 1–45 Hz band, referred as mPDC hereafter, were first compared with the threshold. Then, significant greater (*p* < 0.05) causal interdependence formed an *M* × *M* (*M* = 20 in this study) matrix *B*, where each element *B*_*ij*_ contains the value of the mPDC from the channel *j* to *i*. To display clearly, the weighted digraph is converted into a binary one by applying a threshold. For example, when *B*_*ij*_ exceeds a threshold value, an edge is considered to exist from the node *j* to node *i*. Here, a surrogate data approach was used to obtain the threshold. A 10 s epoch was randomly selected at each channel, and there was no time overlap between two of those 20 channels. Then surrogate *B*_*ij*_ were calculate based on these time-mismatched epochs. This procedure was repeated 10 times. Mean value of the surrogate *B*_*ij*_ was considered as the threshold, which equaled 0.225.

## 3. Results

### 3.1. LZC Analysis in Stroke and Control Groups

**Figure 1:**
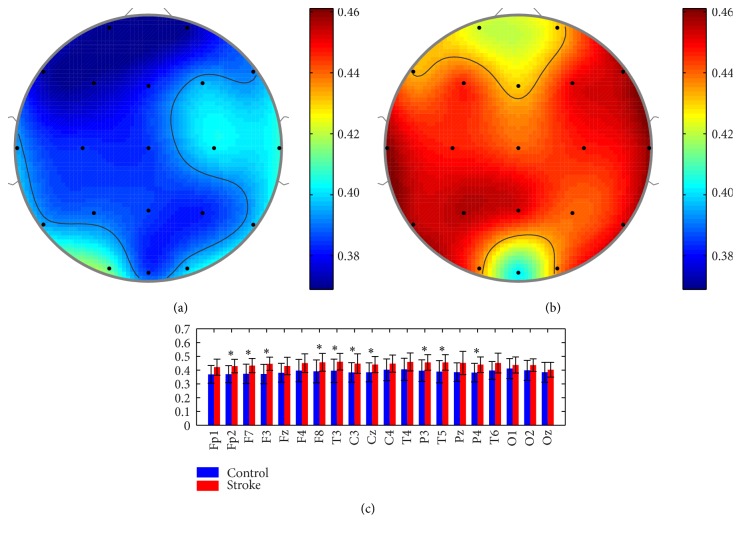
Topographic mapping of the mean LZCs for (a) controls and (b) strokes, as well as (c) the mean value and standard deviation of LZC at each channel. ^*∗*^ *p* < 0.05.

**Figure 2:**
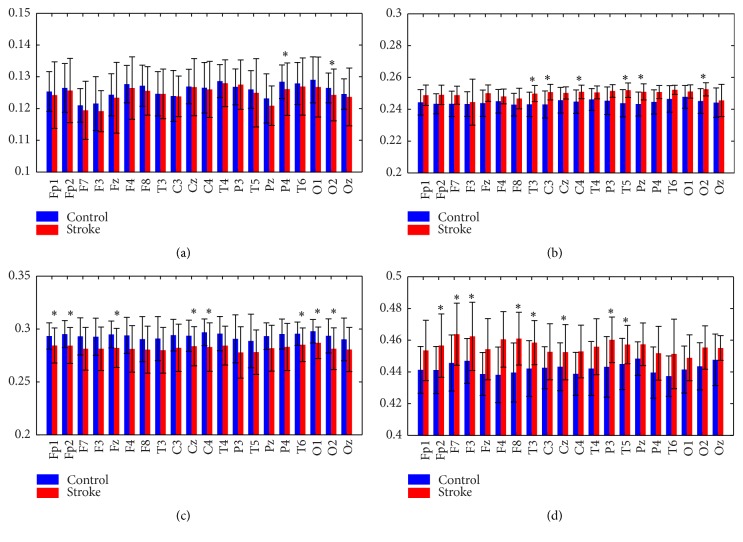
Mean values and standard deviation of LZC in the (a) delta band (1–4 Hz), (b) theta band (4–8 Hz), (c) alpha band (8–13 Hz), and (d) beta band (13–30 Hz). ^*∗*^ *p* < 0.05.

The mean values of the LZC are shown for stoke and control groups in Figure 1. The controls showed lower LZC than the strokes at almost all electrodes. A larger LZC means a greater occurrence chance of new sequence patterns; thus the stroke group had a higher EEG complexity. An independent-samples *t*-test, following the normal distribution test, was performed (^*∗*^ *p* < 0.05). Significant statistical differences were found at channels Fp2, F7, F3, F8, T3, C3, P3, T5, and P4. It should be noted that similar distributions could be observed in both groups. Investigating all the electrodes, we could find higher LZCs at the temporal areas (T4, P4, F4, and C4) in both groups while lower values at prefrontal (Fp1, Fp2) and occipital (O2, Oz) areas. Furthermore, LZC values were also calculated in different EEG frequency bands including delta, theta, alpha, and beta bands, as depicted in Figure 2. It could be observed that the stroke group had higher LZC in theta and beta bands but lower values in delta and alpha bands at all channels. The independent-samples *t*-test showed that both groups significantly differed from each other (^*∗*^ *p* < 0.05) at channels P4 and O2 in delta band, T3, C3, C4, T5, Pz, and O2 in theta band, Fp1, Fp2, Fz, Cz, C4, T6, O1, and O2 in alpha band, and Fp2, F7, F3, F8, Cz, P3, and T5 in beta band.

### 3.2. SampEn Analysis in Stroke and Control Groups

**Figure 3:**
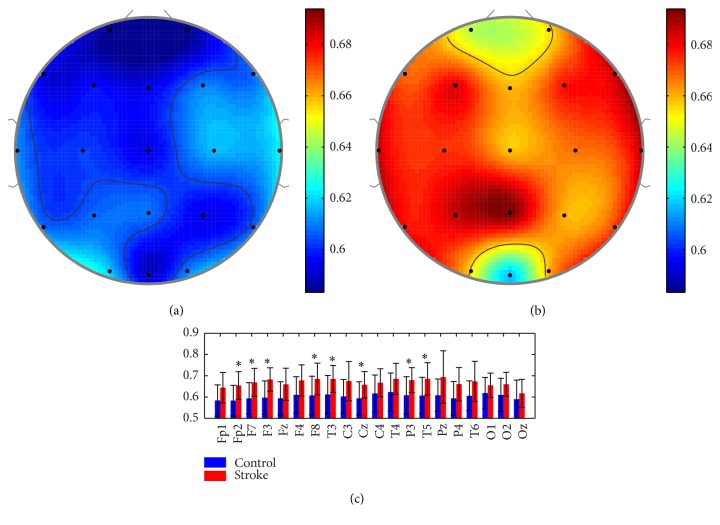
Characteristic distribution of mean SampEn of (a) controls and (b) strokes, as well as (c) the mean value and standard deviation of SampEn in each channel, ^*∗*^ *p* < 0.05.

**Figure 4:**
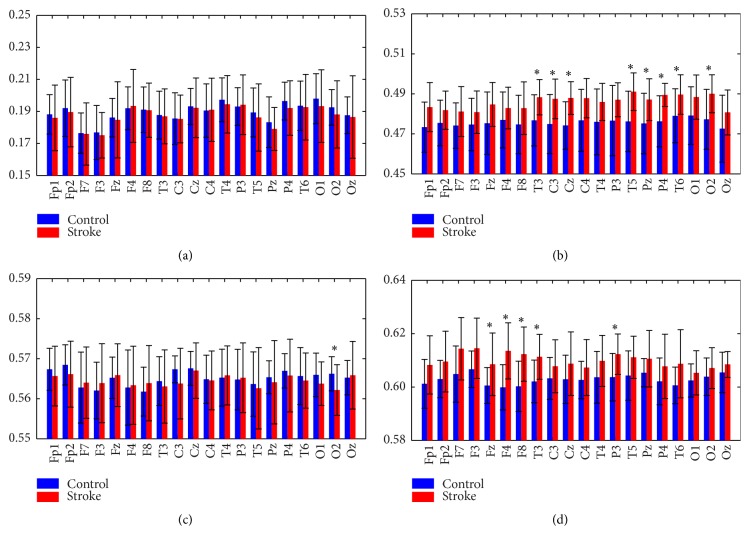
Characteristic distribution of the SampEn in (a) delta, (b) theta, (c) alpha, and (d) beta bands for both groups, ^*∗*^ *p* < 0.05.

As expected, we found similar characteristic distributions between LZC and SampEn; that is, EEG complexity with SampEn was also found to be higher in the stroke group at all electrodes, as shown in Figure 3. SampEn values were computed in delta, theta, alpha, and beta bands, as depicted in Figure 4. Obviously, similar distributions were observed in different bands as LZC.

### 3.3. PDC Analysis in Both Groups

**Figure 5:**
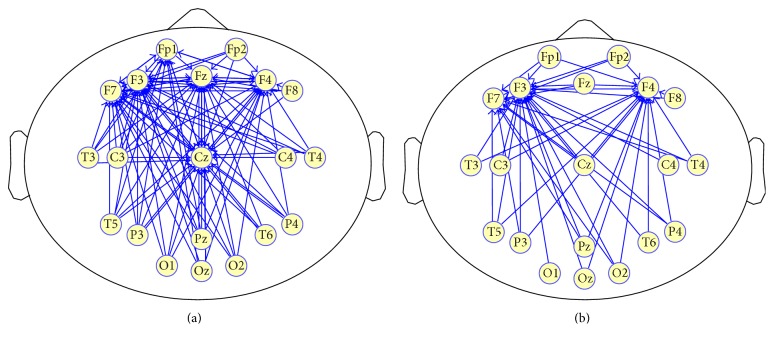
Cortical functional connectivity in the aspect of PDC. Causal interactions with significant causality (connections with mPDC > 0.225 were shown) are presented for controls (a) and strokes (b) under conscious resting conditions.

Figure 5 illustrates the significant cortical functional connectivity (connections with mPDC values greater than 0.225 were shown) for controls (a) and strokes (b), respectively. Compared with the control group, the stroke group displayed a trend of weaker cortical connectivity and a symmetric pattern of functional connectivity; that is, information transmission was found to be lower between electrodes over the brain. This suggests a functional impairment of information transmission in cortical connections in stroke patients.

## 4. Discussions

Stroke is a major cause of adult-onset disability and dependency. Quantified electroencephalography (qEEG) has not been extensively evaluated for its predictive value in stroke recovery, perhaps due to some early disappointing results [38, 39]. More recent reports, however, do suggest a significant predictive value of qEEG for stroke recovery [10, 40]. Finnigan et al. have reported that qEEG measures from acute cortical stroke patients can aid monitoring of brain pathophysiology and perhaps prediction of stroke evolution [13]. The thalamic stroke, however, has not been taken seriously up to now.

In previous studies, power spectrum density within several frequency bands was widely used to analyze the EEGs of patients with stroke, especially in theta and alpha bands, and got some encouraging results [10]. Nonlinear features and functional connectivity of the brain, however, have not been applied to study this disease. In existing studies, LZC has been applied to some cognitive disorders, such as schizophrenia, depression, mild cognitive impairment, and Alzheimer's [18, 19]. Their results showed that both the schizophrenia and the depression groups had a higher LZC (*p* < 0.05) than the controls, while patients with Alzheimer's had a lower LZC. In this paper, results using LZC showed that the stroke patients had higher EEG complexity than that of the controls at all the electrodes. For the SampEn, our results showed higher SampEn values in stroke patients. In previous studies, AD patients were reported to have lower SampEn values than control subjects [41]. Additionally, alpha wave was proven to have a distinct SampEn decrease during the early recovery period after Hypoxic-Ischemic brain injury [26]. In a certain sense, acute thalamic ischemic stroke could be a part of the Hypoxic-Ischemic brain injury, and SampEn values in alpha band were lower in the patients than that in controls as expected in this study. On the other hand, functional connectivity of the brain was well studied in Alzheimer's disease, and the patients displayed a lower connectivity than controls [29]. Similarly, a loss of resting-state functional connectivity was found in patients with thalamic stroke in the acute stage in this study. This is partly because the thalamic stroke was accompanied by loss of neurons, as well as disturbed synaptic transmission. These may lead to abnormal information transmission over the brain.

The progression from ischemia to infarction is a dynamic, rapidly evolving process with irreversible changes occurring within a few minutes to a few hours. EEG may reflect changes in cerebral blood flow and metabolism within seconds as these are directly reflected in the neuronal rhythms. Although the spatial resolution of the EEG is low compared with structural imaging modalities, the high temporal resolution of the EEG may permit a rapid, inexpensive, and sensitive evaluation of instantaneous brain functioning measures for acute cortical stroke patients. EEG analysis could aid monitoring the brain pathophysiology and perhaps predicting the stroke evolution. In recent years, the EEG was testified to carry useful information about the localization of acute cerebral ischemia, but recording densities of 64 channels or higher are required for accurate spatial characterization of focal stroke-related EEG changes.

Although nonlinear EEG analysis and brain network have not been applied as a diagnostic tool yet, our findings demonstrate the possibility of using LZC, SampEn, and PDC to analyze the dynamical behavior and functional connectivity of the brain in patients with acute thalamic stroke. We expect that nonlinear analysis and brain network analysis will provide deeper understandings of the brain function in patients with acute thalamic stroke.

## 5. Conclusion

This study indicates the LZC and SampEn of EEG, as well as the brain functional connectivity, were abnormal in patients with acute thalamic stroke. A higher EEG complexity and weaker brain functional connectivity were obtained in the patients through analysis of LZC, SampEn, and PDC. Additionally, the stroke group had a lower SampEn than the control group in alpha band. These findings show that the nonlinear analysis and brain network analysis may provide a new potential tool to diagnose and monitor the acute thalamic stroke.
